# Hypnotism

**Published:** 1890-06-28

**Authors:** 


### THERAPEUTICS.

### HYPNOTISM.

As was observed to Horatio, "There are more things in heaven and earth than is dreamed of in our philosophy." As regards many matters we yet but "hold the eel of science by the tail," and a little knowledge is often a dangerous thing. It has, however, for years past been the aim and work of some of the greatest thinkers and writers of the day to denude knowledge of its parasites, Ignorance and Credulity. Among other matters we are glad to find that the conditions comprised under the heads of mesmerism, animal magnetism, hypnotism, somnambulism, catalepsy, hallucination, and spiritualism are now being brought within the domain of experimental science. Although the existence of extraordinary phenomena in connection with the above has been known from early times, they have been surrounded by an atmosphere of imposture and delusion. Some forty years ago Dr. Braid, of Manchester, proved that the state known as mesmerism might be caused not only by passes but by straining the eyes on a small object. This, however, Dr. Braid need not have done, for eastern ascetics had long before demonstrated it when looking at the tip of their noses, in the desire to obtain Nirvana. But by Braid's experiments healing effects were produced which a few centuries previously would have been recorded as miracles for the delectation of posterity. Braid also was able to exhalt the faculties and paralyse the will. Thus it is stated that a servant girl being hypnotised, began to repeat passages from the Bible in Hebrew, which she had heard her master read; and a Puritanical old lady was caused to dance a jig! Much more recently the subject was taken up by French medical men at the Hospital of Salpetrière. But it is stated that the supply of a large number of patients suffering from hysteria and other nervous disorders was an essential element for success. This also had been previously demonstrated by Dr. Esdaile, at Madras, who found nervous natives of India very sensible to mesmerism, and Dr. Esdaile performed various operations on these sensitive Indians while they were in the mesmeric sleep. All natives, however, were not susceptible. Liston also performed operations under the mesmeric sleep some 35 years ago.

Mons. Charcot, the Director of Salpetrière, classes the effects produced under two heads, physical and mental or psychial. The latter are connected with will, consciousness, and other phenomena, which we are accustomed to consider mental. The purely physical effects are lethargy, catalepsy, and somnambulism. In lethargy the person appears to be in the deepest sleep. A characteristic feature of this state is, that excitement of muscles causes contraction. Thus if the ulnar nerve is pressed, the third and fourth fingers of the corresponding hand are contracted. Such contractions may also be produced by a magnet not in actual physical contact with the nerve or muscle excited. Lethargy may be made to pass into catalepsy by simply opening the eye-lids. The body now becomes like a lump of plastic material, and may be moulded into any form, which it will retain for a considerable time. When the arm is extended it remains there, showing no tremors of the muscles, as an arm extended voluntarily does. The position in which the body is placed seems to react on the mind, and calls up emotions which are habitually associated with the attitude. Quite as extraordinary are the phenomena of somnambulism, which is produced by animal magnetism or hypnotism. The senses, instead of being asleep, have their sensibility exalted. For instance, subjects feel heat or cold at a distance which would render any change of temperature unappreciable to others.

It is, however, when we come to the phenomena of hallucination and suggestion that the results are, perhaps, most startling. People have been told to look for butterflies where there are no butterflies, and have professed to see butterflies. A girl was told Mr. —— was not present, and could see a hat which somebody placed on Mr. ——'s head, but could not see the gentleman himself. A lady was told to stab another, a paste-board knife was given her, and on awakening she essayed to stab as suggested to her. These were exactly what the mesmerists caused their subjects to do in former days. Persons were told to do such and such an action when they were awake, and did so without being able to give any sufficient reason. We recollect at a mesmeric séance many years ago a girl being told to blow out all the lights in the room, which, on being aroused, she calmly proceeded to do. And we also recollect persons being told to do things even more in the category of things which should be left undone.

Some time back we reviewed Dr. Tuckey's book, entitled, "Psycho-Therapeutics, or Treatment by Sleep and Suggestion." The system adopted by Dr. Tuckey from continental experimentalists, is much as follows: The patient is told to take his place in an arm chair, and to make his mind as much a blank as possible—to think of nothing at all—and to fix his eyes on some special object, as a mark on the ceiling. Then the phenomena, which attend the on-coming of sleep, are gradually suggested to him. "Your sight is growing dim; your eyelids are becoming heavy; numbness is creeping over your limbs; you are getting sleepy; you cannot keep your eyes open." Here the eyes close of themselves, and it is generally found the person is indeed asleep. The patient being thus influenced, the next treatment consists in suggesting an amelioration of morbid conditions. The patient is told that on awakening he will be well, and often is so. In the "Retrospect" for December 14th last, we referred to cases treated by hypnotism and suggestion by Dr. Tuckey. Among the cures reported is a case of insomnia, one of chronic diarrhœa, one of paroxysmal sneezing, one of nocturnal enuresis, one each of functional dysmenorrhœa, tabes dorsalis, torticollis, headache, scriveners' palsy, and dipsomania. We then observed that most of the cases mentioned were of a nervous character, although several were not so. And we particularly instanced the case of chronic diarrhœa, which was said to have lasted since the date of the Crimean war. Chronic diarrhœa, we observed, is attended with certain deposit in the structure of the intestines, of a fatty or lardaceous nature. In fact, there is change of structure. There is certainly such a complaint as nervous diarrhœa, but we can scarcely realise nervous diarrhœa lasting so long without change of structure, and we at present doubt if hypnotism will cure change of structure.

Perhaps, however, the report which will carry most weight is that which has recently proceeded from Leeds, where a series of surgical and dental operations were performed under hypnotic influence induced by Dr. Milne Bramwell, of Goole, Yorkshire. Upwards of sixty medical men and dental surgeons were present. A woman was hypnotised at a word from Dr. Bramwell, and had three teeth extracted without pain. She opened her mouth, spat out, and the like, at word of command, and retired in the somnambulistic state when told to do so. Another operation was opening a large lachrymal abscess, and a third, excision of the tonsil; a fourth, extraction of two teeth from a man; and a fifth, a stump from the jaw of a railway navvy. But the most surprising experiment was the case of a female patient who came with a letter from Dr. Bramwell to Mr. Turner, ordering her to go to sleep, and place herself at Mr. Turner's orders. This patient had sixteen stumps extracted. On the conclusion of the séance, Mr. Pridgin Teal said he "felt sure that the time had now come when we shall have to recognise hypnotism as a necessary part of our study."

Now, we remember many years ago when mesmerism was even more in the atmosphere than hypnotism now is. As before mentioned operations were then performed on patients in the mesmeric condition by Braid, Esdaile, Lister, and others. The recent Leeds hypnotic séance reads in many respects marvellously like the mesmeric séances of the forties of the present century. The more elderly medical men will doubtless recollect the late Dr. Elliotson's connection with mesmerism, and the unfortunate consequences to his professional position. We have, however, become more liberal-minded in these days, and it seems that medical practitioners may become hypnotisers, mesmerists, &c., at their own sweet will, and still retain their professional status. It is well that this should be so. If mesmerism, or its more modern representative, hypnotism, is to be rescued from the hands of adventurers and charlatans, the medical profession must rescue it. If all we hear is correct regarding the doings of the hypnotised after they awake, consequent on suggestions made to them when asleep, we cannot help thinking that the Continental authorities who have forbidden the practice of hypnotism are, to a great extent, right. For there is no reason why crime should not be committed on suggestion, and as a matter of fact it has been so committed. Dr. Tuke has recorded many surprising instances of the influence of the mind on the body. But in hypnotism we not only have the will of the person hypnotised subjected to that of another, but it now seems that the hypnotiser may delegate his power to a third party. We believe that hypnotism may be developed into a great power for good. On the other hand, it may be used so as to cause much evil. Sooner or later we shall be compelled to follow the example of the Continental authorities and legislate on the subject.

